# Revealing Potential Bioactive Compounds and Mechanisms of *Lithospermum erythrorhizon* against COVID-19 via Network Pharmacology Study

**DOI:** 10.3390/cimb44050123

**Published:** 2022-04-19

**Authors:** Ki-Kwang Oh, Md. Adnan

**Affiliations:** Department of Bio-Health Convergence, College of Biomedical Science, Kangwon National University, Chuncheon 24341, Korea; mdadnan@kangwon.ac.kr

**Keywords:** COVID-19, *Lithospermum erythrorhizon*, MAPK signaling pathway, methyl 4-prenyloxycinnamate, tormentic acid, eugenol, RELA, TNF, VEGFA

## Abstract

*Lithospermum erythrorhizon* (LE) is known in Korean traditional medicine for its potent therapeutic effect and antiviral activity. Currently, coronavirus (COVID-19) disease is a developing global pandemic that can cause pneumonia. A precise study of the infection and molecular pathway of COVID-19 is therefore obviously important. The compounds of LE were identified from the Natural Product Activity and Species Source (NPASS) database and screened by SwissADME. The targets interacted with the compounds and were selected using the Similarity Ensemble Approach (SEA) and Swiss Target Prediction (STP) methods. PubChem was used to classify targets linked to COVID-19. The protein–protein interaction (PPI) networks and signaling pathways–targets–bioactive compounds (STB) networks were constructed by RPackage. Lastly, we performed the molecular docking test (MDT) to verify the binding affinity between significant complexes through AutoDock 1.5.6. The Natural Product Activity and Species Source (NPASS) revealed a total of 82 compounds from LE, which interacted with 1262 targets (SEA and STP), and 249 overlapping targets were identified. The 19 final overlapping targets from the 249 targets and 356 COVID-19 targets were ultimately selected. A bubble chart exhibited that inhibition of the MAPK signaling pathway could be a key mechanism of LE on COVID-19. The three key targets (RELA, TNF, and VEGFA) directly related to the MAPK signaling pathway, and methyl 4-prenyloxycinnamate, tormentic acid, and eugenol were related to each target and had the most stable binding affinity. The three bioactive effects on the three key targets might be synergistic effects to alleviate symptoms of COVID-19 infection. Overall, this study shows that LE can play a role in alleviating COVID-19 symptoms, revealing that the three components (bioactive compounds, targets, and mechanism) are the most significant elements of LE against COVID-19. However, the promising mechanism of LE on COVID-19 is only predicted on the basis of mining data; the efficacy of the chemical compounds and the affinity between compounds and the targets in experiment was ignored, which should be further substantiated through clinical trials.

## 1. Introduction

The outbreak of a new health crisis due to coronavirus disease 2019 (COVID-19) occurred in Wuhan, Hubei Province, China, in December 2019 [1]. On 9 January 2020, the novel coronavirus SARS-CoV-2 was officially acknowledged as the sole contributor to this outbreak [2]. The World Health Organization (WHO) initially called this situation a Public Health Emergency of International Concern on 30 January [3], and then announced it was a global pandemic on 11 March [4]. 

The novel coronavirus was designated as severe acute respiratory syndrome coronavirus-2 (SARS-CoV-2, 2019-nCoV) because of its high genetic similarity (around 80%) to SARS-CoV, which caused acute respiratory distress syndrome (ARDS) and high mortality between 2002 and 2003 [5]. The SARS-CoV-2 epidemic was considered to have primarily started through a zoonotic transmission linked to a seafood market in Wuhan, China. Subsequently, it was later identified that human to human transmission played a leading role in the ensuing outbreak [6]. At the time of writing, the COVID-19 has infected 213 countries, and the total number of COVID-19 deaths has reached around 6,130,000 [7]. As of 25 March 2022, approximately 478,260,000 cases were reported worldwide, according to Worldometer [7]. The clinical symptoms of COVID-19 are quite varied, ranging from an asymptomatic condition to intense respiratory distress disorder and multiple organ abnormalities [8]. The common symptoms are fever, dry cough, tachypnea, and shortness of breath [9]. Currently, efforts to developing treatments for COVID-19 by repurposing medications are underway and vaccine development continues. However, the attempts have been hampered by limited information on how this coronavirus penetrates human hosts [10]. Alternatively, natural products have played a vital role in providing drug candidates against various diseases, including the emergence of mutant coronaviruses [11]. Folk remedies have led to the discovery of phytochemicals that are invaluable drug resources and have led to drug candidates such as aspirin from *Salix*, Taxol from *Taxus brevifolia*, and artemisinin from *Artemisia annua* [12,13]. 

The early Korean medical book, *Dongui Bogam*, shows that *Lithospermum erythrorhizon* can be used to treat measles caused by *Measles morbillivirus*. According to an experiment conducted on Beagle dogs, the data suggested that the no observed adverse effect level (NOAEL) of LE extraction was 100 mg/kg/day. Therefore, LE may have a favorable therapeutic effect and is safe to use on virus-related diseases [14]. At present, the bioactive compounds and mechanisms of LE against viruses have not yet been reported. Hence, the exploration of bioactive compounds and mechanisms of LE against the COVID-19 virus should be undertaken to discover more scientific evidence to support its therapeutic application in treating COVID-19. Moreover, we utilized the natural product activity and species source (NPASS) database that we then combined with around 30,000 natural products from diverse traditional and herbal medicines [15]. Moreover, the NPASS is a reliable database with many curated experimental results based on natural compounds [16].

**Figure 1:**
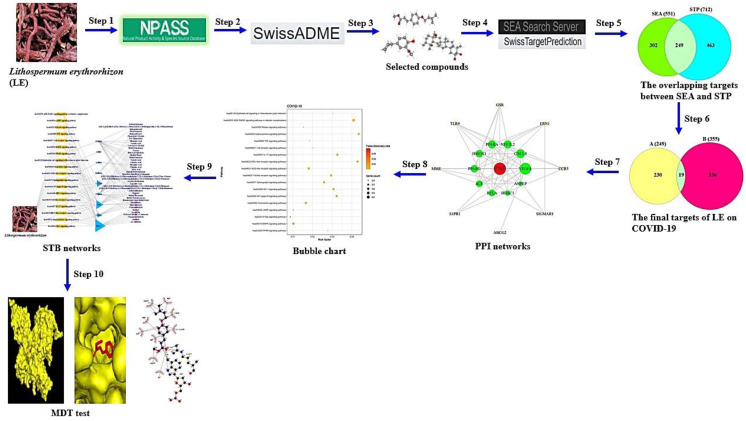
The workflow of this study.

Accordingly, network pharmacology—a multiple analytical mode—can investigate interaction networks such as compounds, genes, protein targets, and diseases [17]. Additionally, network pharmacology can elucidate the mechanism(s) of drug action through networking analysis, which is a role model to shift from “one drug-one target” to “multiple targets” [18]. Therefore, the conception has been extensively utilized to analyze the bioactive compounds and molecular mechanisms of drug candidates against diverse diseases [19]. In this study, network pharmacology was utilized to analyze the bioactive compounds and mechanism(s) of LE against COVID-19. Firstly, compounds from LE were identified using the public database and were confirmed as drug-likeness by the Lipinski rule in SwissADME. Then, targets related to the selected compounds or COVID-19 targets were identified using public databases, and the overlapping targets were selected between compounds and COVID-19 targets. Thirdly, the key bioactive compounds and hub targets of LE against COVID-19 were identified by exploring the interaction of the overlapping targets. Finally, AutoDockTools were used to analyze the binding affinity between promising bioactive compounds and targets. To date, there have been no scientific reports on bioactive compounds and mechanisms of LE against COVID-19. In brief, our study workflow is represented in Figure 1. 

## 2. Materials and Methods

### 2.1. Selective Compounds’ Construction and Drug-Likeness Evaluation

The compound information of LE was collected by NPASS (http://bidd2.nus.edu.sg/NPASS/, accessed on 21 October 2021), Google Scholar, and SMILES (Simplified Molecular Input Line Entry System). The molecular formula of selective compounds was identified using ChemSpider (https://www.chemspider.com/StructureSearch.aspx) (accessed on 21 October 2021) or PubChem (https://pubchem.ncbi.nlm.nih.gov/) (accessed on 21 October 2021) to confirm compound names or structures. The drug-likeness properties of the identified compounds were confirmed through Lipinski’s rule on SwissADME (http://www.swissadme.ch/) (accessed on 21 October 2021) [20]. The compound structures were drawn in PubChem Sketcher V2.4 (https://pubchem.ncbi.nlm.nih.gov/edit3/index.html) (accessed on 21 October 2021) [21]. 

### 2.2. Targets Associated with Selected Compounds or COVID-19

Based on SMILES, targets related to the selected compounds were identified via both SEA (http://sea.bkslab.org/) (accessed on 23 October 2021) [22] and STP (http://www.swisstargetprediction.ch/) (accessed on 24 October 2021) [23] using the “*Homo Sapiens*” mode. We selected the overlapping targets between SEA and STP databases based on the use of a Venn diagram. Moreover, the COVID-19 targets were adapted from PubChem (https://pubchem.ncbi.nlm.nih.gov/) (accessed on 24 October 2021). The final targets between the selected compounds-related targets and COVID-19 targets were visualized by a Venn diagram plotter.

### 2.3. The Analysis of the Protein–Protein Interaction (PPI) Networks

The final targets were utilized to construct a protein–protein interaction (PPI) network on STRING (https://string-db.org/) (accessed on 27 October 2021). In the PPI network, the size of circle represents the degree of value. In particular, a target indicated in red color in the most central position was considered the most significant target.

### 2.4. The Construction of a Bubble Chart

A bubble chart was constructed according to the rich factor, which is defined as the proportion of the number of genes expressed differentially in a signaling pathway [24]. Thus, we identified a hub signaling pathway related to a key target in PPI networks. The bubble chart was plotted in RPackage based on STRING (https://string-db.org/) (accessed on 27 October 2021). 

### 2.5. The Assembly of Signaling Pathways–Targets–Bioactive Compounds (STB) Networks

The STC networks were illustrated the relationships of the three components (signaling pathways–targets–bioactive compounds) and obtained the most critical target in the correlation. In the network, yellow rectangles (nodes) stood for the signaling pathways; blue triangles (nodes) represented targets; and pink circles (nodes) denoted bioactive compounds. The size of the blue triangles marked the number of correlations with the signaling pathways; the size of the pink circles depicted the number of connections with the targets. The merged networks were completed in RPackage. 

### 2.6. The Preparation of the Bioactive Compounds and Targets for Molecular Docking Test (MDT)

The bioactive compounds were associated with the key signaling pathway were extracted .sdf format from PubChem, which were converted into .pdb format using Pymol, and then they were changed into .pdbqt format via AutoDock. The number of three proteins on the MAPK signaling pathway, i.e., TNF (PDB ID: 5YOY), RELA (PDB ID: 2O61), and VEGFA (PDB ID: 3P9W) were obtained using STRING via RCSB PDB (https://www.rcsb.org/) (accessed on 28 October 2021). The targets were converted .pdb format into .pdbqt format via AutoDock (http://autodock.scripps.edu) (accessed on 30 October 2021).

### 2.7. The MDT on a Key Signaling Pathway

The MDT were performed to verify the affinity between bioactive compounds and targets on a key signaling pathway. The set-up condition consisted of a value of 4 for the energy range and a value of 8 for the exhaustiveness as the default settings in order to identify 10 different poses of bioactive compounds. The center value of each target on a key signaling pathway was RELA (x = 15.616, y = −22.641, z = −18.824), TNF (x = 243.718, y = −425.984, z = 261.631), and VEGFA (x = −12.652, y = 70.481, z = −40.286). The cubic box size of active site was set at x = 40 Å, y = 40 Å, and z = 40 Å. The 2D molecular docking studies were performed in LigPlot^+^ 2.2 [25]. The lower the binding energy (the higher the negative value), the greater the stable binding is between the bioactive and the target. 

## 3. Results 

### 3.1. Potential Bioactive Compounds from LE

We obtained the 82 bioactive compounds in LE through NPASS database and physicochemical properties of these compounds are listed in Table 1. All of them were confirmed by Lipinski’s rule [26] and TPSA (<140 Å²) [27]. Thus, we considered that these compounds might be potential therapeutic agents against COVID-19. 

### 3.2. Targets Associated with the 82 Compounds or COVID-19

**Figure 2:**
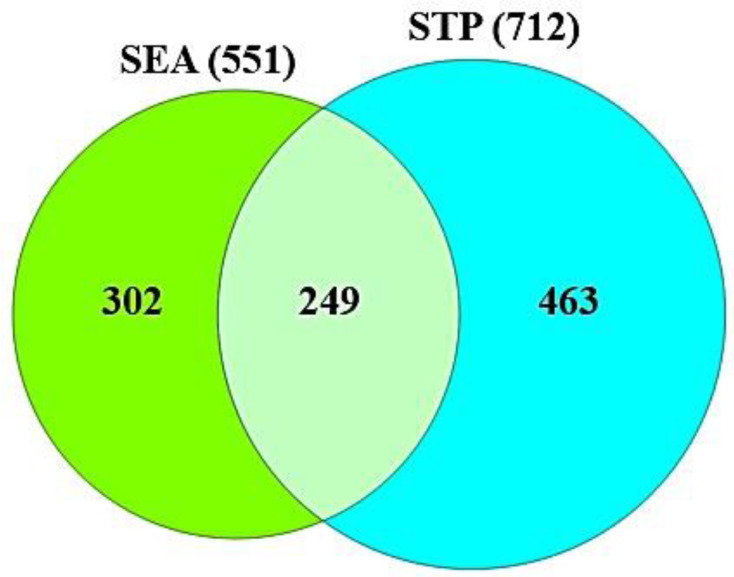
The overlapping 249 targets between 550 from SEA and 712 targets from STP.

**Figure 3:**
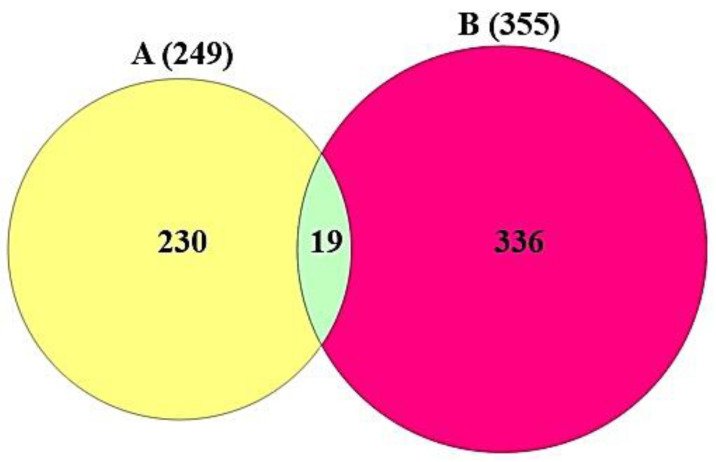
The final 19 targets between the 249 targets and 355 COVID-19 targets.

As shown in Supplementary Table S1, the total number of 1262 targets (SEA + STP) related to 82 compounds was identified in DisGeNET and OMIM. Then, the overlapping 249 targets between SEA and STP were obtained (Figure 2). The 249 targets (Supplementary Table S2) were analyzed with 356 COVID-19 related targets (Supplementary Table S3). Finally, the Venn diagram (Figure 3) showed that 19 overlapping targets (Supplementary Table S4) were directly associated with response to COVID-19 infection.

### 3.3. The Protein–Protein Interaction (PPI) Networks from 19 Targets

**Figure 4:**
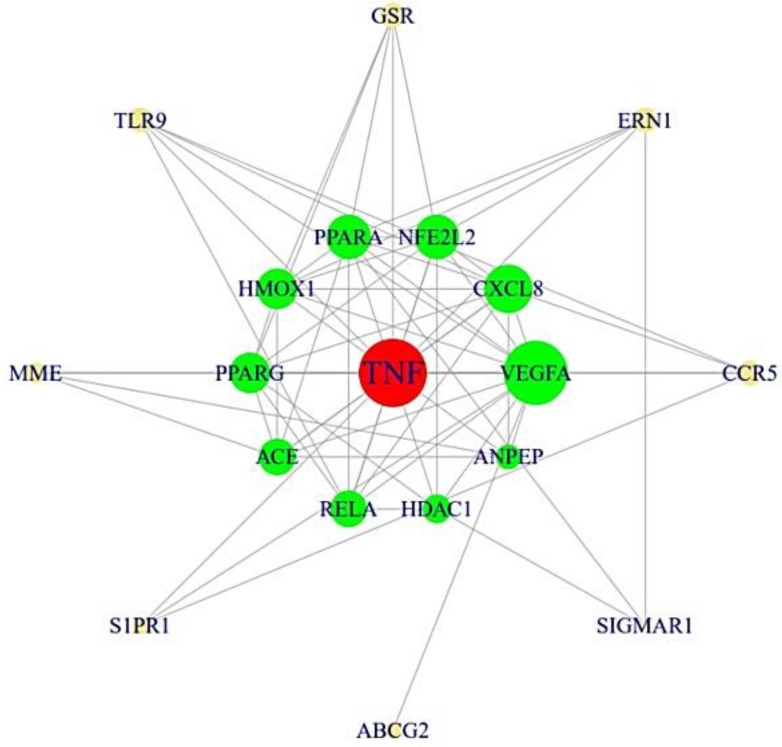
The PPI networks (19 nodes and 69 edges).

In the PPI networks, the TNF target was considered to be the most significant target with the highest degree of value (15) (Table 2). Moreover, the 19 targets were closely interconnected with each other (19 nodes and 69 edges) (Figure 4). The TNF in the most central position was the most significant target in the PPI networks.

### 3.4. A Bubble Plot and Signaling Pathways–Targets–Bioactive Compounds (STB) Networks

**Figure 5:**
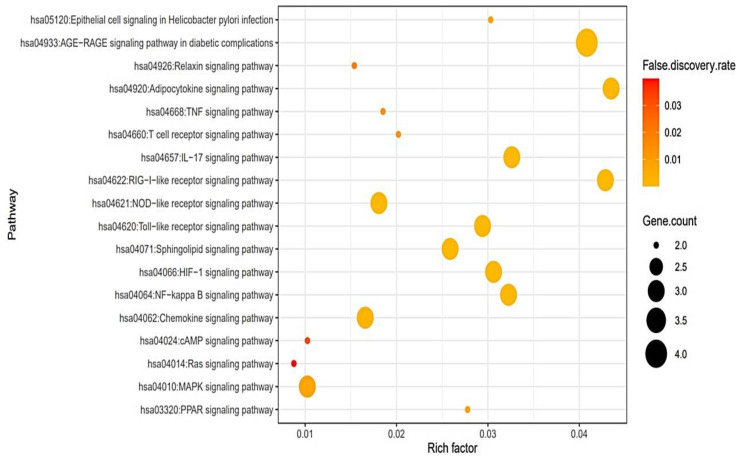
A bubble plot of 18 signaling pathways related to the response to LE bioactive compounds against COVID-19.

**Figure 6:**
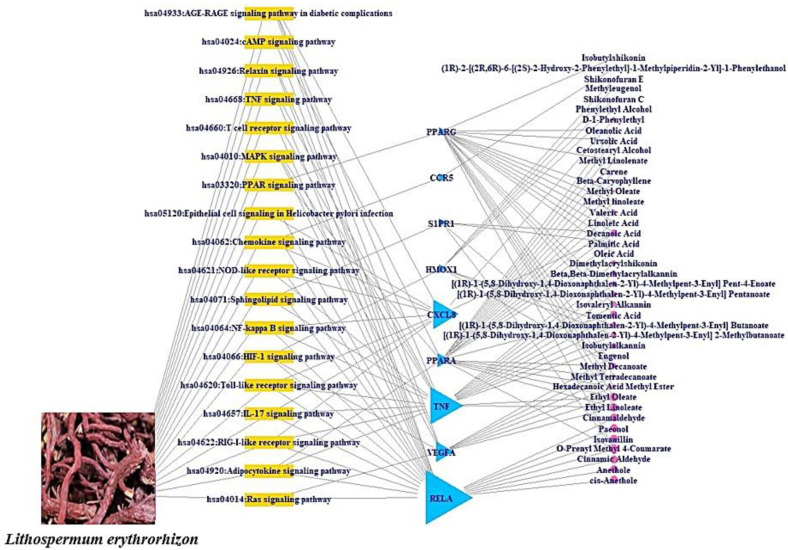
The STB networks. Yellow triangle: signaling pathway; blue sky triangle: target; pink circle: LE bioactive compound.

The Kyoto Encyclopedia of Genes and Genomes (KEGG) pathway enrichment analysis indicated that the 19 targets were associated directly with 18 signaling pathways (False Discovery Rate < 0.05) (Figure 5). The identified 18 signaling pathways were involved in the response to a COVID-19 infection. Detailed information on the 18 signaling pathways is presented in Table 3. Additionally, a bubble plot suggested that the RAS signaling pathway might be a key signaling pathway due to the lowest rich factor (0.008). Moreover, we performed an STB networks analysis to identify the most important target based on the degree of value (Figure 6). Thus, the highest degree of value in STB networks was the RELA target with 17 degrees, which was considered as a notable target against COVID-19 (Table 4). Comprehensively, a signaling pathway that combined both TNF (a key target in PPI networks) and RELA (a key target in STB networks) was the MAPK signaling pathway, which had an antagonistic propensity on the relatively lower rich factor found in the 18 signaling pathways. We observed that the uppermost signaling pathway was not the RAS signaling pathway but the MAPK signaling pathway, which consisted of the two core targets (TNF and RELA).

### 3.5. The Molecular Docking Test on MAPK Signaling Pathway against COVID-19

**Figure 7:**
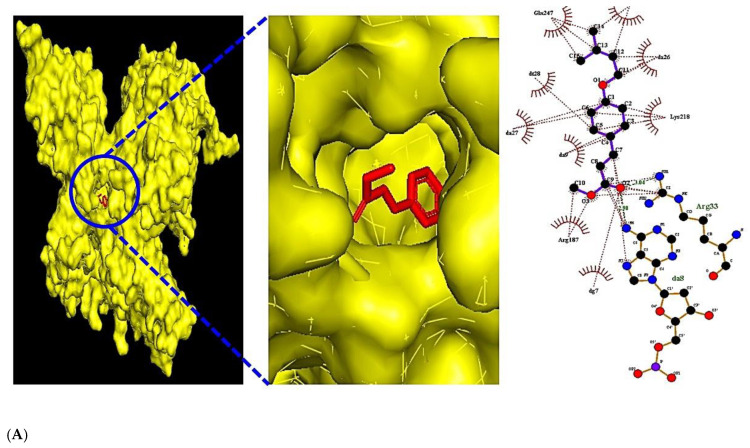

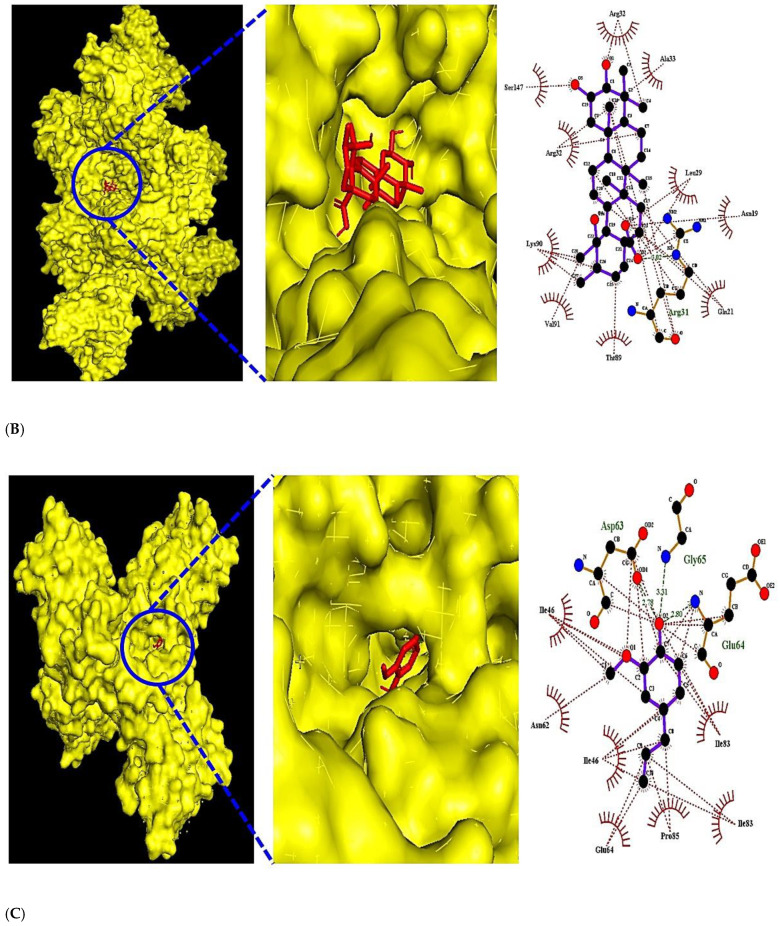
(**A**) The MDT of Methyl 4-prenyloxycinnmate (PubChem ID: 14414116) on RELA (PDB ID: 2O61). (**B**) The MDT of Tormentic acid (PubChem ID: 73193) on TNF (PDB ID: 5YOY). (**C**) The MDT of Eugenol (PubChem ID: 3314) on VEGFA (PDB ID: 3P9W).

The potential bioactive compounds were docked against three targets (RELA, TNF, and VEGFA) to measure the binding energy. The MDT of the affinity between R1–R5 and RELA target (PDB ID: 2O61) in the “*Homo sapiens*” setting was analyzed. The binding energy of R1-RELA, R2-RELA, R3-RELA, R4-RELA, and R5-RELA demonstrated at −7.1, −6.2, −5.7, −5.5, and −5.4 kcal/mol, respectively (Table 5). Methyl 4-prenyloxycinnamate (R1) had the strongest affinity for RELA (PDB ID: 2O61) (Figure 7A). The binding energy between T1–T12 and TNF target (PDB ID: 5YOY) in the “*Homo sapiens*” setting was revealed. The presented binding energy of T1-TNF, T2-TNF, T3-TNF, T4-TNF, T5-TNF, T6-TNF, T7-TNF, T8-TNF, T9-TNF, T10-TNF, T11-TNF, and T12-TNF were exposed to −7.3, −7.1, −6.6, −6.5, −6.4, −6.3, −6.3, −6.3, −6.2, −6.1, −5.6 and −5.0 kcal/mol, respectively (Table 6). Tormentic acid (T1) manifested the strongest affinity for VEGFA (PDB ID: 3P9W) (Figure 7B). The binding energy between V1–V6 and VEGFA target in the “*Homo sapiens*” setting was uncovered. The presented binding energies of V1-VEGFA, V2-VEGFA, V3-VEGFA, V4-VEGFA, V5-VEGFA, and V6-VEGFA were −6.1, −5.2, −4.7, −4.6, −4.2, and −3.7 kcal/mol, respectively (Table 7). Eugenol (V1) exhibited the strongest affinity for VEGFA (Figure 7C). Collectively, methyl 4-prenyloxycinnamate, tormentic acid, and eugenol of LE on COVID-19 were promising bioactive compounds to dampen MAPK signaling pathway.

## 4. Discussion

The compounds–targets network indicated that LE compounds might be significant ligands that could be used to alleviate COVID-19 symptoms. Of these, RELA has a more significant effect than any other kinds of targets. Furthermore, based on the pathway enrichment, the MAPK signaling pathway is the uppermost mechanism of LE against COVID-19. Thus, three targets (RELA, TNF, and VEGFA) linked to the MAPK signaling pathway might be promising targets for use against COVID-19. Accordingly, the docking score on the three targets suggested that three bioactive compounds (methyl 4-prenylcinnamate, tormentic acid, and eugenol) were considered as the most notable compounds of LE against COVID-19. Meanwhile, the results of the KEGG pathway enrichment analysis showed that nine targets might play important roles against COVID-19. The relationships of the 18 signaling pathways with anti-virus were discussed as follows.

- AGE-RAGE signaling pathway in diabetic complications: Activation of the binding of AGE to its receptor RAGE can stimulate cytokine production, can cause tissue damage, and the suppression of AGE-RAGE can effectively reduce inflammation [28].
- Adipocytokine signaling pathway: Most adipocytokines are pro-inflammatory factors and they are closely linked to chronic inflammation [29,30].
- RIG-I-like receptor (RLR) signaling pathway: The RIG-I-like receptors (RLRs), RIG-I, MDA5, and LGP2, play an important role in pathogen recognition of RNA virus infections that instigate and regulate antiviral immunity [31].
- IL-17 signaling pathway: IL-17 can play vital roles in responding to pathogenicity in diverse tissues, as well as being important for inflammation balance and tissue cohesion during viral attacks [32].
- Toll-like receptor (TLR) signaling pathway: Nucleic acids originating from bacteria and viruses can be recognized by the intracellular Toll-like receptor (TLR) and they are also sensitive to self-nucleic acids in disease conditions such as autoimmunity [33].
- HIF-1 signaling pathway: The dysfunction of HIF-1α develops influenza A virus (IAV) replication by triggering autophagy in alveolar epithelial cells [27].
- NF-κb (Nuclear Factor kappa-light-chain-enhancer of activated B cells) signaling pathway: Viruses have evolved to exploit NF-κb-driven cellular functions, and the understanding of molecular mechanisms might be a new strategy against viral diseases [34].
- Sphingolipid signaling pathway: Sphingolipid metabolites, such as ceramide and sphingosine-1-phosphate, are signaling messengers that tune a wide range of cellular processes and are essential for immunity, inflammation, and inflammatory disorders [35].
- NOD-like receptor (NLR) signaling pathway: NLRs have been linked to human diseases, including infections, inflammatory disorders, and even chronic inflammation [36].
- Chemokine signaling pathway: In COVID-19 patients, inhibiting the secretion of cytokines and chemokines dulled the cytokine storm that represented the severity of the disease and was a negative side effect [37].
- PPAR (Peroxisome Proliferator-Activated Receptor) signaling pathway: The regulation of PPAR-α (Peroxisome Proliferator-Activated Receptor-alpha) with agonists enhanced herpesvirus replication and reactive oxygen species (ROS) production [38].
- MAPK (Mitogen-Activated Protein Kinase) signaling pathway: it was reported that the virus’s existence in hosts could activate the MAPK signaling pathway; some viral specific proteins can maintain the persistent activation of the MAPK signaling pathway [39].
- T cell receptor (TCR) signaling pathway: The TCR recognizes pathogens on major histocompatibility complex molecules with the cooperation of CD4 (Cluster of Differentiation 4) or CD8 (Cluster of Differentiation 8) co-receptors and produces cytokines [40].
- TNF (Tumor necrosis factor-alpha) signaling pathway: TNF-α boosters influenza A virus-induced production of antiviral cytokines by activating RIG-I (Retinoic acid-inducible gene I) gene expression [41].
- Relaxin signaling pathway: Relaxin receptor abnormality enhances vascular inflammation and damages external remodeling in arteriovenous fistulas [42].
- cAMP (Cyclic Adenosine MonoPhosphate) signaling pathway: cAMP stimulates interleukin-10 production as the anti-inflammatory cytokine [43].
- RAS (Renin-Angiotensin System) signaling pathway: The use of RAS antagonists might increase the risk of developing a SARS-CoV-2 infection. However, it is not sufficient evidence for discontinuing RAS blockers in patients with hypertension [44].

Based on the pathway enrichment analysis, RELA was considered as a hub target in LE against COVID-19. The RELA was directly enriched in 17 out of 18 signaling pathways by the MAPK signaling pathway, indicating that the MAPK signaling pathway might be a hub signaling pathway in LE against COVID-19. The other two targets (TNF and VEGFA) that are directly related to the MAPK signaling pathway might be important targets for creating synergistic effects against COVID-19. A report demonstrated that inhibition of RELA of the NF-κB component reduce cytokine production and thus could alleviate inflammation severity [45]. Most recently, it has been reported that anti-TNF treatment for COVID-19 patients with rheumatoid arthritis diseases showed preventive effects against the high levels of cytokines involved in the immune response of infection, and the therapeutic application of anti-TNFs can lessen the incidence of severe inflammation of COVID-19 [46]. Notably, a report indicated that vascular endothelial growth factor A (VEGFA) antagonized by angiotensin-converting enzyme 2 (ACE2) that is upregulated by COVID-19 infection because COVID-19 inhibits the expression of ACE2. Consequently, VEGFA increases vascular permeability and lessens endothelial damage [47].

**Figure 8:**
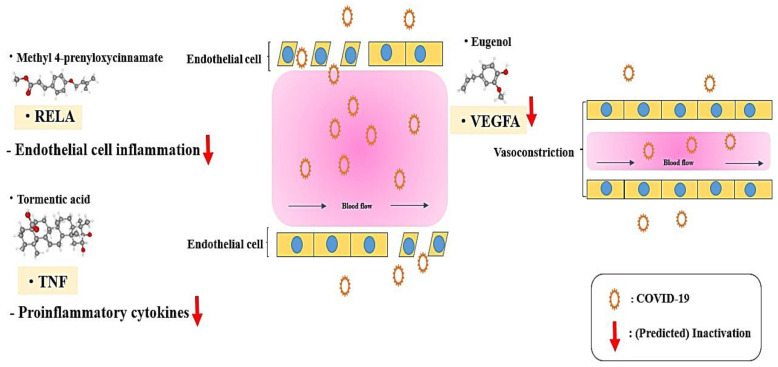
The potential mechanism effectors of LE against COVID-19.

Endothelial cell inflammation is a serious symptom of COVID-19 infection, and its uncontrollable cytokine production in tissues and cells causes a severe immune reaction, which is defined as a “cytokine storm” that results in aggravating pneumonia. Moreover, inhibition of the other two targets (TNF and VEGFA) related to the MAPK signaling pathway contribute to anti-proinflammation and anti-vascular permeability against COVID-19. Therefore, the key mechanism of LE against COVID-19 might be the ability to block inflammation and vascular permeability in tissues and/or cells by inactivating the MAPK signaling pathway (Figure 8). However, this research still has some limitations. The incompleteness of the natural products dataset might create a fallacy and COVID-19 data is updated continually as a new version. During the analysis, some results might cause an error unexpectedly, if we are only focused on computational methods.

Thus, we need to validate the pharmacological mechanism via in vitro or in vivo tests. Thereby, the usefulness of computational approach might be represented. As a matter of fact, network pharmacology is a holistic perspective to search for multiple factors against specific diseases, which might provide significant clues about the relationships between components such as signaling pathways, targets, and compounds.

## 5. Conclusions

The bioactive compounds and mechanism(s) of LE against COVID-19 were first explored using network pharmacology. The findings of this study suggest that the final bioactive compounds were methyl 4-prenylcinnamate, tormentic acid, and eugenol and their targets were RELA, TNF, and VEGFA, respectively. The mechanism(s) of LE against COVID-19 might inhibit cell inflammation and permeability against COVID-19 by inactivating the MAPK signaling pathway. This research provides a scientific indication to support LE’s therapeutic effects on COVID-19, and thus, the proper application of these three bioactive compounds against COVID-19 might lead to promising synergistic effects such as anti-inflammation and anti-permeability in order to alleviate COVID-19 symptoms. In parallel, this study needs to be further verified through in vitro or in vivo models.

## Figures and Tables

**Table 1:** The physicochemical properties of 82 chemical compounds in LE.

	Compounds		Lipinski Rules						
		PubChem ID	MW	HBA	HBD	MLogP	Lipinski’s Violations	Bioavailability Score	TPSA
No.			<500	<10	≤5	≤4.15	≤1	>0.1	<140 Å²
1	(S)-1-Phenylethanol	443135	122.16	1	1	1.87	0	0.55	20.23
2	3-Methylbutanoic acid	10430	102.13	2	1	0.89	0	0.85	37.30
3	cis-Caffeic acid	1549111	180.16	4	3	0.70	0	0.55	77.76
4	Phenylethyl alcohol	6054	122.16	1	1	1.87	0	0.55	20.23
5	Thiophene	8030	84.14	0	0	1.12	0	0.55	28.24
6	Caryophyllene	5281515	204.35	0	0	4.63	1	0.55	0.00
7	Alkannin	72521	288.30	5	3	0.42	0	0.55	94.83
8	b-b-Dimethylacrylalkannin	442720	370.40	6	2	1.43	0	0.55	100.90
9	Shikonofuran C	5321288	358.43	5	2	2.36	0	0.55	79.90
10	Isovanillin	12127	152.15	3	1	0.51	0	0.55	46.53
11	(R)-2-methylbutanoate	6950479	102.13	2	1	0.89	0	0.85	37.30
12	Ethyl oleate	5363269	310.51	2	0	5.03	1	0.55	26.30
13	Camphor	2537	152.23	1	0	2.30	0	0.55	17.07
14	(−)-Caryophyllene oxide	1742210	220.35	1	0	3.67	0	0.55	3.67
15	Methyl linolenate	5319706	292.46	2	0	4.61	1	0.55	26.30
16	Totarol	92783	286.45	1	1	4.92	1	0.55	20.23
17	Oleanolic acid	10494	456.70	3	2	5.82	1	0.85	57.53
18	(S)-2-methylbutanoate	6950480	101.12	2	0	0.89	0	0.85	40.13
19	Hexadecanoic acid methyl ester	8181	270.45	2	0	4.44	1	0.55	26.30
20	(−)-Borneol	1201518	154.25	1	1	2.45	0	0.55	20.23
21	Cinnamic aldehyde	637511	132.16	1	0	2.01	0	0.55	17.07
22	Valeric acid	7991	102.13	2	1	0.89	0	0.85	37.30
23	Methyl 4-prenyloxycinnmate	14414116	246.30	3	0	3.48	0	0.55	35.53
24	(3S,4S)-4,7,7-trimethylbicyclo[2.2.1]heptan-3-ol	12242815	154.25	1	1	2.45	0	0.55	20.23
25	Paeonol	11092	166.17	3	1	0.83	0	0.55	46.53
26	β-Selinene	519361	204.35	0	0	4.63	1	0.55	0.00
27	Docosanol	12620	326.60	1	1	5.84	1	0.55	20.23
28	Phenylacetaldehyde	998	120.15	1	0	1.78	0	0.55	17.07
29	11-O-Acetylalkannin	137628887	330.33	6	2	0.82	0	0.55	100.90
30	palmitic acid	985	256.42	2	1	4.19	1	0.85	37.30
31	furfural	7362	96.08	2	0	−0.56	0	0.55	30.21
32	isobutyric acid	6590	88.11	2	1	0.49	0	0.85	37.30
33	isobutylshikonin	479500	358.39	6	2	1.28	0	0.55	100.90
34	Shikalkin	5208	288.30	5	3	0.42	0	0.55	94.83
35	shikonin	479503	288.30	5	3	0.42	0	0.55	94.83
36	Propionylshikonin	153984	344.36	6	2	1.06	0	0.55	100.90
37	β-hydroxyisovalerylshikonin	479502	388.41	7	3	0.71	0	0.55	121.13
38	eugenol	3314	164.20	2	1	2.01	0	0.55	29.46
39	cis-Anethole	1549040	148.20	1	0	2.67	0	0.55	9.23
40	tormentic Acid	73193	488.70	5	4	4.14	0	0.55	97.99
41	oleic Acid	445639	282.46	2	1	4.57	1	0.85	37.30
42	1-eicosanol	12404	298.55	1	1	5.39	1	0.55	20.23
43	decanoic acid	2969	172.26	2	1	2.58	0	0.85	37.30
44	borneol	64685	154.25	1	1	2.45	0	0.55	20.23
45	ethyl linoleate	5282184	308.50	2	0	4.93	1	0.55	26.30
46	cetostearyl alcohol	62238	512.93	2	2	7.28	2	0.17	40.46
47	2-acetylpyrrole	14079	109.13	1	1	−0.18	0	0.55	32.86
48	(1R)-2-[(2R,6R)-6-[(2S)-2-hydroxy-2-phenylethyl]-1-methylpiperidin-2-yl]-1-phenylethanol	6604328	339.47	3	2	3.03	0	0.55	43.70
49	methyleugenol	7127	178.23	2	0	2.30	0	0.55	18.46
50	caffeic acid	689043	180.16	4	3	0.70	0	0.55	77.76
51	β-Ionone	638014	192.30	1	0	2.94	0	0.55	17.07
52	carene	26049	136.23	0	0	4.29	1	0.55	0.00
53	dimethylacrylshikonin	479499	370.40	6	2	1.43	0	0.55	100.90
54	acetylshikonin	479501	330.33	6	2	0.82	0	0.55	100.90
55	methyl tetradecanoate	31284	242.40	2	0	3.94	0	0.55	26.30
56	deoxyshikonin	98914	272.30	4	2	1.25	0	0.55	74.60
57	buthylshikonin	10089766	358.39	6	2	1.28	0	0.55	100.90
58	3-methylbut-2-enoic Acid	10931	100.12	2	1	0.79	0	0.85	37.30
59	shikonofuran E	5321290	356.41	5	2	2.28	0	0.55	79.90
60	methyl oleate	5364509	296.49	2	0	4.80	1	0.55	26.30
61	Isovalerylshikonin	479497	372.41	6	2	1.51	0	0.55	100.90
62	α-methyl-butylshikonin	479498	372.41	6	2	1.51	0	0.55	100.90
63	isobutylalkannin	137629300	358.39	6	2	1.28	0	0.55	100.90
64	Methyl linoleate	5284421	294.47	2	0	4.70	1	0.55	26.30
65	(−)-camphor	444294	152.23	1	0	2.30	0	0.55	17.07
66	isovaleryl alkannin	5318685	372.41	6	2	1.51	0	0.55	100.90
67	2-pentylfuran	19602	138.21	1	0	1.84	0	0.55	13.14
68	[(1R)-1-(5,8-dihydroxy-1,4-dioxonaphthalen-2-yl)-4-methylpent-3-enyl] (6Z,9Z)-octadeca-6,9-dienoate	44438574	550.73	6	2	3.95	1	0.55	100.90
69	[(1R)-1-(5,8-dihydroxy-1,4-dioxonaphthalen-2-yl)-4-methylpent-3-enyl] pent-4-enoate	9999214	370.40	6	2	1.43	0	0.55	100.90
70	[(1R)-1-(5,8-dihydroxy-1,4-dioxonaphthalen-2-yl)-4-methylpent-3-enyl] benzoate	10475609	392.40	6	2	1.99	0	0.55	100.90
71	[(1R)-1-(5,8-dihydroxy-1,4-dioxonaphthalen-2-yl)-4-methylpent-3-enyl] pentanoate	145992534	476.61	6	2	2.15	0	0.55	151.50
72	nonanal	31289	142.24	1	0	2.39	0	0.55	17.07
73	ursolic acid	64945	456.70	3	2	5.82	1	0.85	57.53
74	methyl decanoate	8050	186.29	2	0	2.87	0	0.55	26.30
75	hexanal	6184	100.16	1	0	1.39	0	0.55	17.07
76	2-methylbutanoic acid	8314	102.13	2	1	0.89	0	0.85	37.30
77	shikonofuran D	5321289	344.40	5	2	2.14	0	0.55	79.90
78	linoleic acid	5280450	280.45	2	1	4.47	1	0.85	37.30
79	D-1-phenylethyl	637516	122.16	1	1	1.87	0	0.55	20.23
80	P-cymene	7463	134.22	0	0	4.47	1	0.55	0.00
81	phenanthrene	995	178.23	0	0	5.17	1	0.55	0.00
82	anethole	637563	148.20	1	0	2.67	0	0.55	9.23

**Table 2:** The degree value of targets in PPI networks.

No.	Target	Degree of Values
1	TNF	16
2	VEGFA	15
3	CXCL8	11
4	NFE2L2	10
5	PPARA	10
6	HMOX1	9
7	PPARG	9
8	ACE	8
9	RELA	8
10	HDAC1	6
11	ANPEP	5
12	CCR5	5
13	ERN1	5
14	GSR	5
15	TLR9	5
16	MME	4
17	S1PR1	3
18	SIGMAR1	3
19	ABCG2	1

**Table 3:** Targets in 18 signaling pathways with enrichment related to COVID-19.

KEGG ID	Description	Target Genes	False Discovery Rate
hsa04933	AGE-RAGE signaling pathway in diabetic complications	RELA, TNF, CXCL8, VEGFA	0.000067
hsa04920	Adipocytokine signaling pathway	RELA, TNF, PPARA	0.000440
hsa04622	RIG-I-like receptor signaling pathway	RELA, TNF, CXCL8	0.000440
hsa04657	IL-17 signaling pathway	RELA, TNF, CXCL8	0.000760
hsa04620	Toll-like receptor signaling pathway	RELA, TNF, CXCL8	0.000760
hsa04066	HIF-1 signaling pathway	RELA, HMOX1, VEGFA	0.000760
hsa04064	NF-kappa B signaling pathway	RELA, TNF, CXCL8	0.000760
hsa04071	Sphingolipid signaling pathway	RELA, TNF, S1PR1	0.000950
hsa04621	NOD-like receptor signaling pathway	RELA, TNF, CXCL8	0.000950
hsa04062	Chemokine signaling pathway	RELA, CXCL8, CCR5	0.002100
hsa05120	Epithelial cell signaling in Helicobacter pylori infection	RELA, CXCL8	0.005700
hsa03320	PPAR signaling pathway	PPARA, PPARG	0.006400
hsa04010	MAPK signaling pathway	RELA, TNF, VEGFA	0.007200
hsa04660	T cell receptor signaling pathway	RELA, TNF	0.010200
hsa04668	TNF signaling pathway	RELA, TNF	0.011800
hsa04926	Relaxin signaling pathway	RELA, VEGFA	0.016500
hsa04024	cAMP signaling pathway	RELA, PPARA	0.031200
hsa04014	Ras signaling pathway	RELA, VEGFA	0.039900

**Table 4:** The degree value of targets in the STB networks.

No.	Target	Degree of Values
1	RELA	17
2	TNF	11
3	CXCL8	8
4	VEGFA	5
5	PPARA	3
6	PPARG	2
7	CCR5	2
8	HMOX1	1
9	S1PR1	1
10	NFE2L2	0
11	ACE	0
12	HDAC1	0
13	ANPEP	0
14	ERN1	0
15	GSR	0
16	TLR9	0
17	MME	0
18	SIGMAR1	0
19	ABCG2	0

**Table 5:** Binding energy of potential active compounds on RELA (PDB ID: 2O61).

					Grid Box		Hydrogen Bond Interactions	Hydrophobic Interactions
Protein	Ligand	PubChem ID	Symbol	Binding Energy (kcal/mol)	Center	Dimension	Amino Acid Residue	Amino Acid Residue
RELA (PDB ID: 2O61)	Methyl 4-prenyloxycinnmate	14414116	R1	−7.1	x = 15.616	size_x = 40	Arg33	Gln247, Lys218, Arg187
					y = −22.641	size_y = 40		
					z = −18.824	size_z = 40		
	Paeonol	11092	R2	−6.2	x = 15.616	size_x = 40	Arg246	Lys272, Lys241
					y = −22.641	size_y = 40		
					z = −18.824	size_z = 40		
	Isovanillin	12127	R3	−5.7	x = 15.616	size_x = 40	N/A	Arg33, Arg187, Lys218
					y = −22.641	size_y = 40		
					z = −18.824	size_z = 40		
	Anethole	637563	R4	−5.5	x = 15.616	size_x = 40	Arg305	Val248, Lys218, Arg246
					y = −22.641	size_y = 40		Gln247, Phe307
					z = −18.824	size_z = 40		
	Cinnamic aldehyde	637511	R5	−5.4	x = 15.616	size_x = 40	N/A	Pro189, Asp185, Cys120
					y = −22.641	size_y = 40		His88, Tyr36, Leu154
					z = −18.824	size_z = 40		Val121, Asn155, Ala188

**Table 6:** Binding energy of potential active compounds on TNF (PDB ID: 5YOY).

	`				Grid Box		Hydrogen Bond Interactions	Hydrophobic Interactions
Protein	Ligand	PubChem ID	Symbol	Binding Energy (kcal/mol)	Center	Dimension	Amino Acid Residue	Amino Acid Residue
TNF (PDB ID: 5YOY)	Tormentic acid	73193	T1	−7.3	x = 243.718	size_x = 40	Arg31	Arg32, Ala33, Leu29
					y = −425.984	size_y = 40		Asn19, Gln21, Thr89
					z = 261.631	size_z = 40		Val91, Lys90, Arg32
								Ser147
	[(1R)-1-(5,8-dihydroxy-1,4-dioxonaphthalen-2-yl)-4-methylpent-3-enyl] pent-4-enoate	9999214	T2	−7.1	x = 243.718	size_x = 40	Asn30	Lys128, Arg31, Ala84,
					y = −425.984	size_y = 40		Leu29, Arg82, Tyr87
					z = 261.631	size_z = 40		Gln27, Trp28, Asn46
								Asp45, Leu43, Glu127
	Dimethylacrylshikonin	479499	T3	−6.6	x = 243.718	size_x = 40	Trp94, Phe144	Gln21, Ala145, Gly105
					y = −425.984	size_y = 40		Lys65, Asp143, Pro20
					z = 261.631	size_z = 40		
	Isovalerylshikonin	479497	T4	−6.5	x = 243.718	size_x = 40	Asn93, Phe144	Pro20, Gln21, Gly105
					y = −425.984	size_y = 40		Lys65, Asp143. Ala145
					z = 261.631	size_z = 40		Trp94
	Isobutylalkannin	137629300	T5	−6.4	x = 243.718	size_x = 40	Thr69. Tyr60, Ser85	Lys65, Gly66, Phe68
					y = −425.984	size_y = 40		Arg67, Gly66, Lys58
					z = 261.631	size_z = 40		
	α-Methyl-butylshikonin	479498	T6	−6.3	x = 243.718	size_x = 40	Ala33, Ala145	Val17, Arg32, Ala18
					y = −425.984	size_y = 40		Pro20, Gln21, Glu146
					z = 261.631	size_z = 40		Arg31, Val91, Ser147
	Isobutylalkannin	137629300	T7	−6.3	x = 243.718	size_x = 40	Thr69, Tyr60, Ser85	Lys65, Gly66, Phe68
					y = −425.984	size_y = 40		Arg67, Lys58
					z = 261.631	size_z = 40		
	β-β-Dimethylacrylalkannin	442720	T8	−6.3	x = 243.718	size_x = 40	Ser56, Asp54, Tyr53	His73, Leu75, Pro113
					y = −425.984	size_y = 40	Gln67, Lys65	Asn57,Tyr115
					z = 261.631	size_z = 40		
	Buthylshikonin	10089766	T9	−6.2	x = 243.718	size_x = 40	Thr79, Ser95, Gln149	Lys90, Asn92, Ser81
					y = −425.984	size_y = 40		Glu146, Ile97, Thr77
					z = 261.631	size_z = 40		Asn137, Ile136, Glu135
								His78
	[(1R)-1-(5,8-dihydroxy-1,4-dioxonaphthalen-2-yl)-4-methylpent-3-enyl] pentanoate	145992534	T10	−6.1	x = 243.718	size_x = 40	Thr69, Tyr60	Gly66, Lys65, Lys58
					y = −425.984	size_y = 40		Arg67, Ser85, Phe68
					z = 261.631	size_z = 40		
	Ethyl oleate	5363269	T11	−5.6	x = 243.718	size_x = 40	N/A	Glu127, Arg82, Leu36
					y = −425.984	size_y = 40		Ala35, Leu36, Gln125
					z = 261.631	size_z = 40		Arg31, Asn34, Arg32
								Ala35, Gln125, Asn34
	Ethyl linoleate	5282184	T12	−5.0	x = 243.718	size_x = 40	N/A	Gly101, Tyr53, Asn57
					y = −425.984	size_y = 40		Ser56, His73, Leu75
					z = 261.631	size_z = 40		Pro113, Ala111, Ser52
								Ala33, Gln67

**Table 7:** Binding energy of potential active compounds on VEGFA (PDB ID: 3P9W).

					Grid Box		Hydrogen Bond Interactions	Hydrophobic Interactions
Protein	Ligand	PubChem ID	Symbol	Binding Energy (kcal/mol)	Center	Dimension	Amino Acid Residue	Amino Acid Residue
VEGFA (PDB ID: 3P9W)	Eugenol	3314	V1	−6.1	x = −12.652	x = 40	Asp63, Gly65, Glu64	Ile83, Pro85, Glu64
					y = 70.481	y = 40		Ile46, Asn62, Ile46
					z = −40.286	z = 40		
	Ethyl linoleate	5282184	V2	−5.2	x = −12.652	x = 40	N/A	Ile46, Pro85, His86
					y = 70.481	y = 40		Phe36, Ser50, Cys60
					z = −40.286	z = 40		Asp34, Glu64
	Methyl tetradecanoate	31284	V3	−4.7	x = −12.652	x = 40	Glu64	Ile46, Pro85, Asp63
					y = 70.481	y = 40		His86, Phe36, Ser50
					z = −40.286	z = 40		Asn62, Phe47, Asp63
								Ile83
	Ethyl oleate	5363269	V4	−4.6	x = −12.652	x = 40	N/A	Ile83, Pro85, Glu64
					y = 70.481	y = 40		Asn62, Asp63, Glu64
					z = −40.286	z = 40		His86, Ile46, Asp63
								Ile83, Pro85
	Hexadecanoic acid methyl ester	8181	V5	−4.2	x = −12.652	x = 40	N/A	Ile46, Ile83, Glu64
					y = 70.481	y = 40		Phe36, His86, Ser50
					z = −40.286	z = 40		Cys61, Asn62, Pro85
	Methyl decanoate	8050	V6	−3.7	x = −12.652	x = 40	N/A	Cys68, Asp63, Phe47
					y = 70.481	y = 40		Ile46, Ser50, Phe36
					z = −40.286	z = 40		Asp34,His86, Glu64
								Glu67

## Data Availability

All data generated or analyzed during this study are included in this published article (and its Supplementary Information files).

## Supplementary Materials

The following supporting information can be downloaded at: https://www.mdpi.com/article/10.3390/cimb44050123/s1, Table S1: SEA (Similarity Ensemble Approach): 550 targets; STP (SwissTargetPrediction): 712 targets. Table S2: The overlapping targets between SEA and STP: 249 targets. Table S3: COVID 19-related targets: 356 targets. Table S4: The final 19 overlapping targets related to COVID-19.

## Abbreviations

ACE2	Angiotensin Converting Enzyme 2
AGE-RAGE	Advanced Glycation Endproduct–Receptor for Advanced Glycation Endproduct
cAMP	Cyclic Adenosine MonoPhosphate
CD4	Cluster of Differentiation 4
CD8	Cluster of Differentiation 8
COVID-19	Coronavirus disease 2019
HIF-1	Hypoxia-inducible factor 1
HIF-1α	Hypoxia-inducible factor 1α
IL-17	Interleukin 17
KEGG	Kyoto Encyclopedia of Genes and Genomes
LE	Lithospermum erythrorhizon
LGP2	Laboratory of Genetics and Physiology 2
MAPK	Mitogen-Activated Protein Kinase
MDA5	Melanoma Differentiation-Associated protein 5
NF-κb	Nuclear Factor kappa-light-chain-enhancer of activated B cells
NLRs	NOD-like receptors
NPASS	Natural Product Activity and Species Source
PPAR	Peroxisome Proliferator-Activated Receptor
PPAR-α	Peroxisome Proliferator Activated Receptor Alpha
RAS	Renin-Angiotensin System
RELA	v-rel avian reticuloendotheliosis viral oncogene homolog A
RIG-I	Retinoic acid-inducible gene I
RLRs	RIG-I-like receptors
RNA	Ribonucleic acid
ROS	Reactive Oxygen Species
SARS-CoV-2	Severe Acute Respiratory Syndrome Coronavirus 2
SEA	Similarity Ensemble Approach
SMILES	Simplified Molecular Input Line Entry System
STP	Swiss Target Prediction
TCR	T Cell Receptor
TLR	Toll-like Receptor
TNF	Tumor Necrosis Factor
TNF-α	Tumor Necrosis Factor Alpha
VEGFA	Vascular Endothelial Growth Factor A
